# Supplementary material for: Complexome profiling of the Chlamydomonas psb28 mutant reveals TEF5 as an early PSII assembly factor
Source: Plant Cell. 2025 Mar 17;37(6):koaf055. doi: 10.1093/plcell/koaf055 (PMC12164590; doi:10.1093/plcell/koaf055)
Supplement: koaf055_Supplementary_Data [file koaf055_supplementary_data.zip › psb28_interactive.zip!/plots/intensity/qualityPlots/peptides/HLA8_Cre01.g048150.t1.2.html]

### Cre01.g048150.t1.2

Trivial name: HLA8  
SeqCoverage [median]: 9.00  
Mass: 30.47 kDa  
  
 

| Sequence | MissedCleavage | Mass [Da] | wtIntensity | muIntensity | Score |
| --- | --- | --- | --- | --- | --- |
| DYIPAEDAAQAAR | 0 | 1389.65 | 0.00 | 968.90 | 74.84 |
| VVDGDTLVINGER | 0 | 1385.72 | 823.30 | 1402.55 | 141.93 |
|  |  |  | 823.3 | 2371.4 |  |
